# Protocol for high-power, brain-focused microwave fixation to define rodent metabolism

**DOI:** 10.1016/j.xpro.2025.103794

**Published:** 2025-04-25

**Authors:** Alex R. Cantrell, Tara R. Hawkinson, Jelena A. Juras, Madison B. Webb, Terrymar Medina, Roberto A. Ribas, James Collins, Amelia A. Bunnell, Lyndsay E.A. Young, Kia H. Markussen, Douglas A. Andres, Joanna R. Long, Craig W. Vander Kooi, Bret N. Smith, Ramon C. Sun, Matthew S. Gentry

**Affiliations:** 1 Department of Biochemistry and Molecular Biology, University of Florida, College of Medicine, Gainesville, FL 32610, USA; 2 Department of Molecular and Cellular Biochemistry, University of Kentucky, College of Medicine, Lexington, KY 40536, USA; 3 Department of Cell and Molecular Pharmacology and Experimental Therapeutics, Medical University of South Carolina, Charlston, SC 29425, USA; 4 Department of Biomedical Sciences, Colorado State University, Fort Collins, CO 80523, USA; 5 Center for Advanced Spatial Biomolecule Research, University of Florida, College of Medicine, Gainesville, FL 32610, USA

**Keywords:** metabolism, metabolomics, neuroscience

## Abstract

Analysis of metabolites provides key insights into brain physiology and function. Due to post-mortem metabolism, both the euthanasia method and dissection time can make a critical difference. Here, we describe a protocol to euthanize rodents by microwave irradiation. This workflow details steps for animal placement, tissue fixation, and post-fixation processing. This protocol enables the rapid halting of metabolic activity for the accurate assessment of the metabolome *in situ* for analyses such as mass spectrometry and nuclear magnetic resonance.

For complete details on the use and execution of this protocol, please refer to Juras et al.^1^

## Highlights

• Instructions for securing rodents in appropriate holder
• Procedures for safely operating and optimizing microwave apparatus
• Guidance on post-fixation processing

## Abstract (editor highlights)

Publisher’s note: Undertaking any experimental protocol requires adherence to local institutional guidelines for laboratory safety and ethics.

## Graphical abstract

**Figure:**
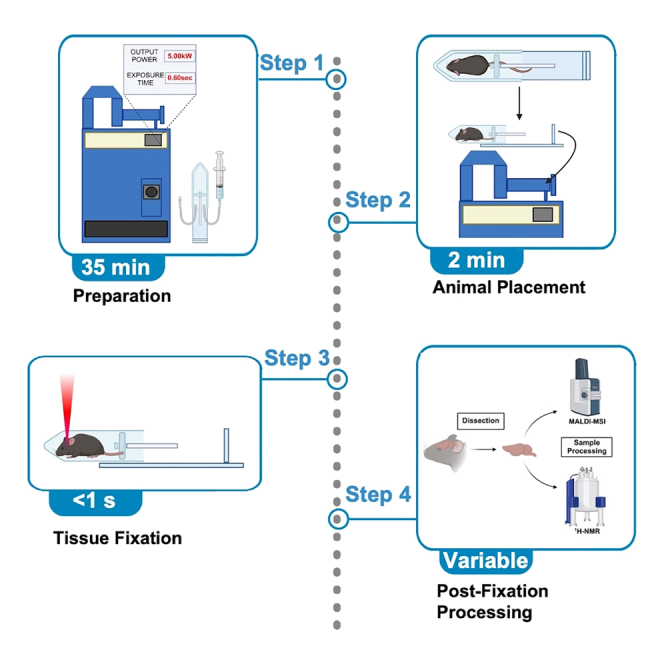

## Before you begin

Herein, we describe a protocol utilizing microwave irradiation for euthanasia of rodents and rapid fixation of brain tissue for accurate metabolomic analyses. Before starting the protocol, there are a few items to consider. First, high-power, brain-focused microwave irradiation is an approved euthanasia technique by the American Veterinary Medical Association that rapidly causes animals to lose consciousness (<100 ms) and euthanizes them (<1 s).^2^ According to the manufacturer, instrument settings and animal placement will only affect the fixation quality and not the rapid euthanasia time. Second, for the installation of a high-power, brain-focused microwave, power outlets must be configured to meet power requirements of 190–240 V 3-A 3 Phase or 380–440 V 20 A 3 Phase prior to operation. Finally, the mouse genotype, weight, lipid composition, and water content all impact the fixation outcome. Additionally, the output of the magnetron within the microwave will vary slightly between instruments. Therefore, it is advised that optimization is performed for each mouse model prior to any experiments.

***Note:*** For the purposes of optimizing microwave settings, here are the recommended exposure times for a power at 5 kW by the manufacturer: Mouse (20–30 g; 0.94 s), Rat (150–250 g; 1.70 s), Rat (250–400 g; 1.40 s), Rat (400–500 g; 1.45 s).

***Note:*** The following protocol only describes how to use microwave fixation for brain metabolism. Using microwave fixation to fix peripheral organs requires additional optimization.

**CRITICAL:** The microwave should only be operated with an animal loaded in the chamber. Firing the microwave while unloaded is a fire hazard.

**CRITICAL:** Metal cannot be used in the microwave as it is a fire hazard. Animals containing metal implants (e.g., surgical implants, ear tags, etc.) should not undergo microwave euthanasia.

### Institutional permissions

The Institutional Animal Care and Use Committees at the University of Kentucky and at the University of Florida have approved all animal procedures utilized in this study. Before employing this protocol, readers should acquire training to use the high-powered microwave and obtain appropriate permission from the relevant governing bodies at their respective institutions.

### Preparation of the high-power, focused microwave

**Timing: 30 min**

The microwave must preheat prior to being used for euthanizing the first animal of the cohort.

1. Turn on the microwave and allow 30 min prior to use for the microwave to prime and preheat.
2. After 30 min, set the parameters of the microwave to the predetermined optimum power and time.

***Note:*** These are the only parameters that can be adjusted. These settings (power = 5 kW; time = 0.6 s) were determined to provide optimal fixation of tissue from mice—weighing 20–25 g—used in this experiment.

### Preparation of animal holder

**Timing: 5 min**

Follow these steps to choose the appropriate applicator head and animal holder for the animals being used and to fill the water jacket on the animal holder.

**Figure 1:**
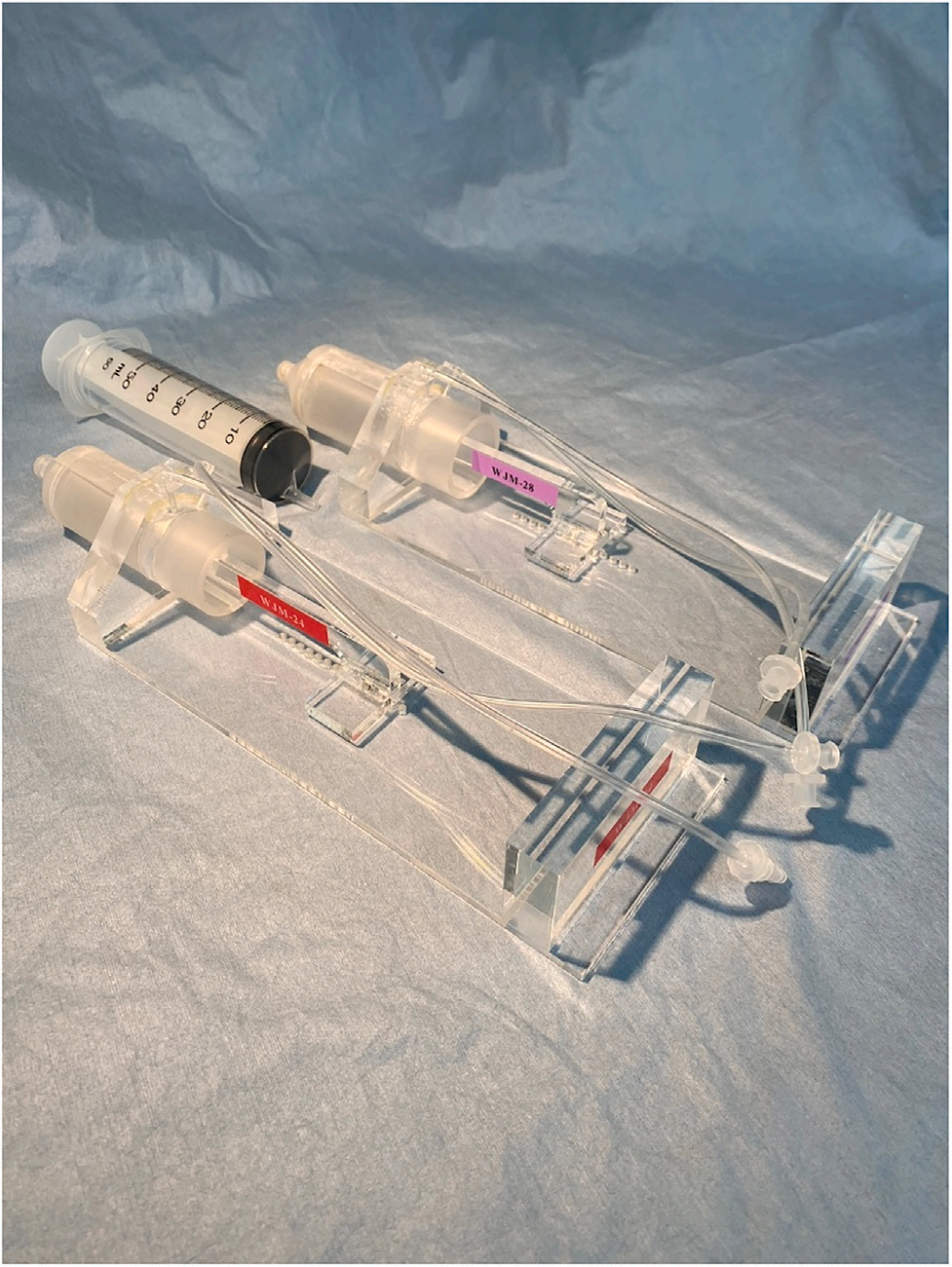
Animal holders for different sized mice WJM-24 (left, for 15–20 g mice) and WJM-28 (right, for 20–40 g mice) Holders are also available for rats and requires the appropriate applicator head to be installed for accurate function.

3. Choose the appropriate applicator head and animal holder (Figure 1):
  a. TAW-174P: used for WJM-24 (15–20 g mouse), WJM-28 (20–40 g mouse), or WJM-30 (40–50 g mouse) animal holders.
  b. TAW-424SP: used for WJR-S (150–250 g rat) animal holder.
  c. TAW-424MP: used for WJR-M (250–400 g rat), WJR-L (400–500 g rat) animal holders.
4. Remove the male lure fitting from the female fitting at the end of the tubing.
5. Fill the water jacket surrounding the animal holder with deionized water by attaching a syringe onto the polyetheretherketone (PEEK) tubing, and carefully flush water through the line. Ensure that no bubbles are trapped in the holder, and no water is leaking around the edges.
6. Replace the male fitting to prevent water leaking from the tube and remove the syringe.

**CRITICAL:** This step is extremely important as the water is used to ensure even heat transfer across the target tissue during the fixation process. Water must be replaced after each animal.

## Key resources table

**Table:** 

REAGENT or RESOURCE	SOURCE	IDENTIFIER
**Chemicals, peptides, and recombinant proteins**		

Ethanol	Decon Labs	CAS#: 64-17-5

**Experimental models: Organisms/strains**		

Mouse: C57BL/6J (6–8 months, males)	The Jackson Laboratory	JAX: 000664

**Other**		

High power-focused microwave	Muromachi Kikai Company	MMW-05
Animal holder	Muromachi Kikai Company	WJM-24 (for 15-20g Mouse), WJM-28 (for 20-40g Mouse), WJM-30 (for 40-50g Mouse), WJR-S (for 150-250g Rat), WJR-M (for 250-400g Rat), WJR-L (for 400-500g Rat)
Applicator head	Muromachi Kikai Company	TAW-174P (for WJM-24, WJM-28, and WJM-30), TAW-424SP (for WJR-S), TAW-424MP (for WJR-M and WJR-L)
60 mL syringe only, Luer lock tip	BD Biosciences	BD 309653
Over-the-head ear muffs	Fisher Scientific	19-130-1978
Safety glasses	Fisher Scientific	19-181-514

## Step-by-step method details

### Placing the animal into the holder

**Timing: 1 min**

**Figure 2:**
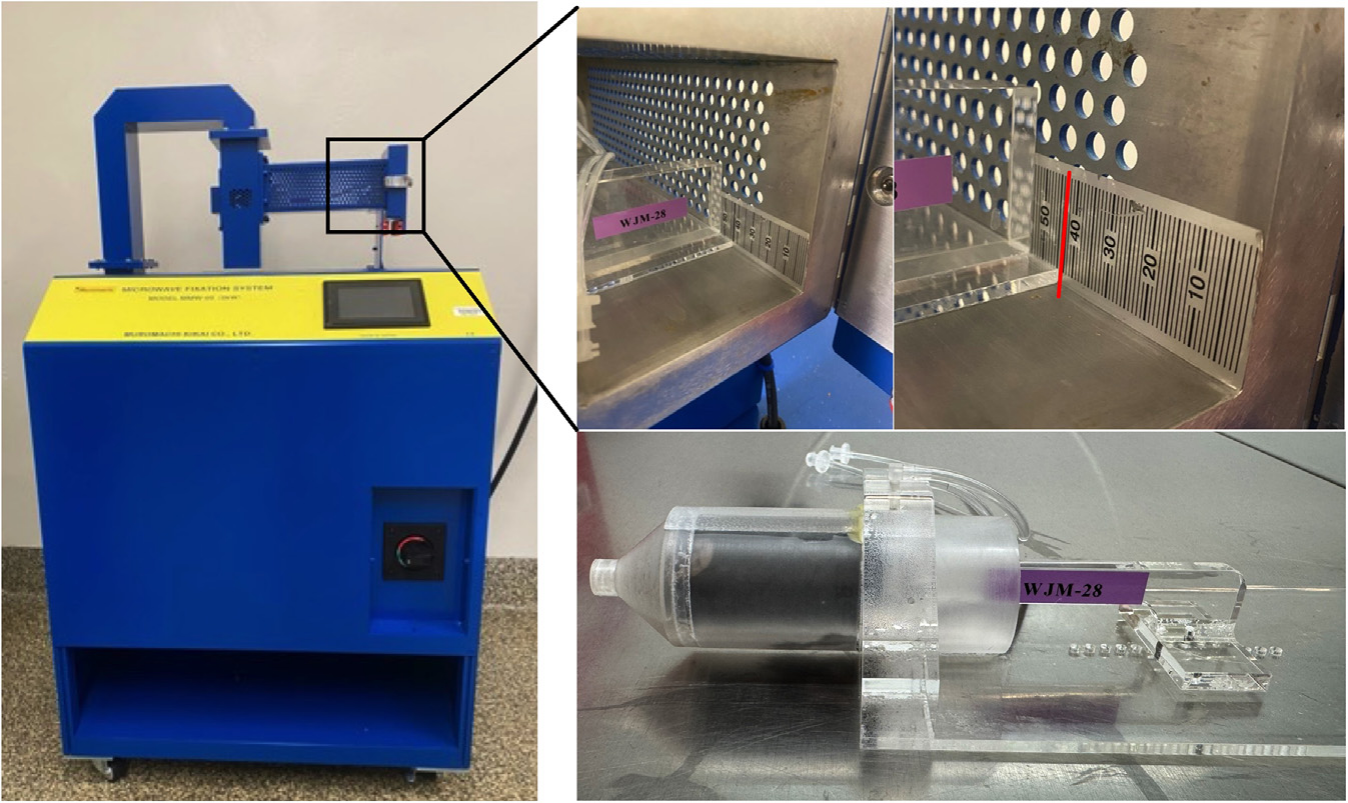
Placement of the mouse in animal holder and the holder in the applicator head of the Muromachi Kikai Company Microwave Model MMW-05 For this system, the recommended depth is 43 mm in the applicator head.

Here, we describe how to properly secure the animal in the size-appropriate holder (Figure 2).

1. Restrain the animal by scruffing.
2. Guide the animal’s snout into the animal holder towards the cone shaped end.
3. Secure the animal in the apparatus with the holder plunger, ensuring the animal’s tail is visible. Make sure the animal is fully restrained without being too tight or too loose.

***Note:*** Animal positioning in the holder and in the microwave will only affect fixation quality according to the manufacturer. The mouse will be euthanized very rapidly when placed inside the microwave.

**CRITICAL:** Ensure the mouse cannot move back and forth once it is placed in the holder, as the microwave beam is focused to a very specific target. If the mouse can move, then one cannot guarantee proper fixation will occur.

### Place the animal into the high-power, brain-focused microwave

**Timing: 1 min**

To place the animal holder within the microwave follow these steps.

***Note:*** Misplacement of the animal holder will result in under- or over-fixation of the brain.

4. Following the markings on the interior of the microwave, gently slide the animal holder inside the microwave (Figure 2).

***Note:*** The manufacturer recommends placing the animal holder 43 mm in the applicator head. Placement may require some optimization if tissue fixation is insufficient.

5. Close the metal door on the microwave and latch the door.
6. Once the door is shut, press “Next” on the screen and “OK” to confirm settings.
7. Press START twice in rapid succession and wait until FINISHED appears.
8. Unlatch the door, remove the plastic holder from the microwave, and carefully remove the animal from the holder.

**CRITICAL:** Both the animal and the holder are hot after the microwave procedure. Leave the animal in the holder at room temperature for 5 min before beginning the dissection. Do not worry about metabolite degradation in the brain, as the brain has been fixed.

### Dissection

**Timing:** 2 min

These steps describe removing the animal from the holder and dissecting the brain.

***Note:*** The animal will physically contract due to the microwave fixation.

9. After the animal has sufficiently cooled, remove the animal and stretch the animal out.
10. To remove the brain, first decapitate the animal using scissors.
11. Pull the skin back to reveal the skull.
12. Using surgical scissors, cut along the sides of the skull or down the midline and peel the skull back to expose the brain.

**CRITICAL:** Inspect the brain to ensure proper fixation. If the brain is under-fixed, it will still have a wet appearance and will exhibit greater elasticity. If the brain is over-fixed, it will be brittle. Properly fixed tissue should be firm, yet compressible. Color of the tissue is also an indication of fixation quality.^3^ The color will be red/pink if under-fixed and brown if fixed appropriately.

13. Carefully remove the brain from the base of the skull using a spatula.
14. Store tissue at −80°C until use.

### Microwave re-charging

**Timing: 3–10 min**

Here, we describe preparing the microwave for the subsequent animals.

***Note:*** The microwave takes 3 min between animals to re-charge to the appropriate power.

15. To obtain consistency between animals, approximately 10 min is needed between the euthanasia of one animal and beginning the process for the next animal.

### Cleaning the animal holder between animals

**Timing: 2 min**

These steps describe proper cleaning of the animal holders between euthanizations.

***Note:*** The animal holder must be cleaned and prepared between each animal.

16. Cleaning should be done with 70% ethanol. Spray the animal holder and thoroughly wipe it down with a paper towel and Kimwipe.
17. Replace the water in the animal holder with fresh deionized water and repeat the process.

## Expected outcomes

**Figure 3:**
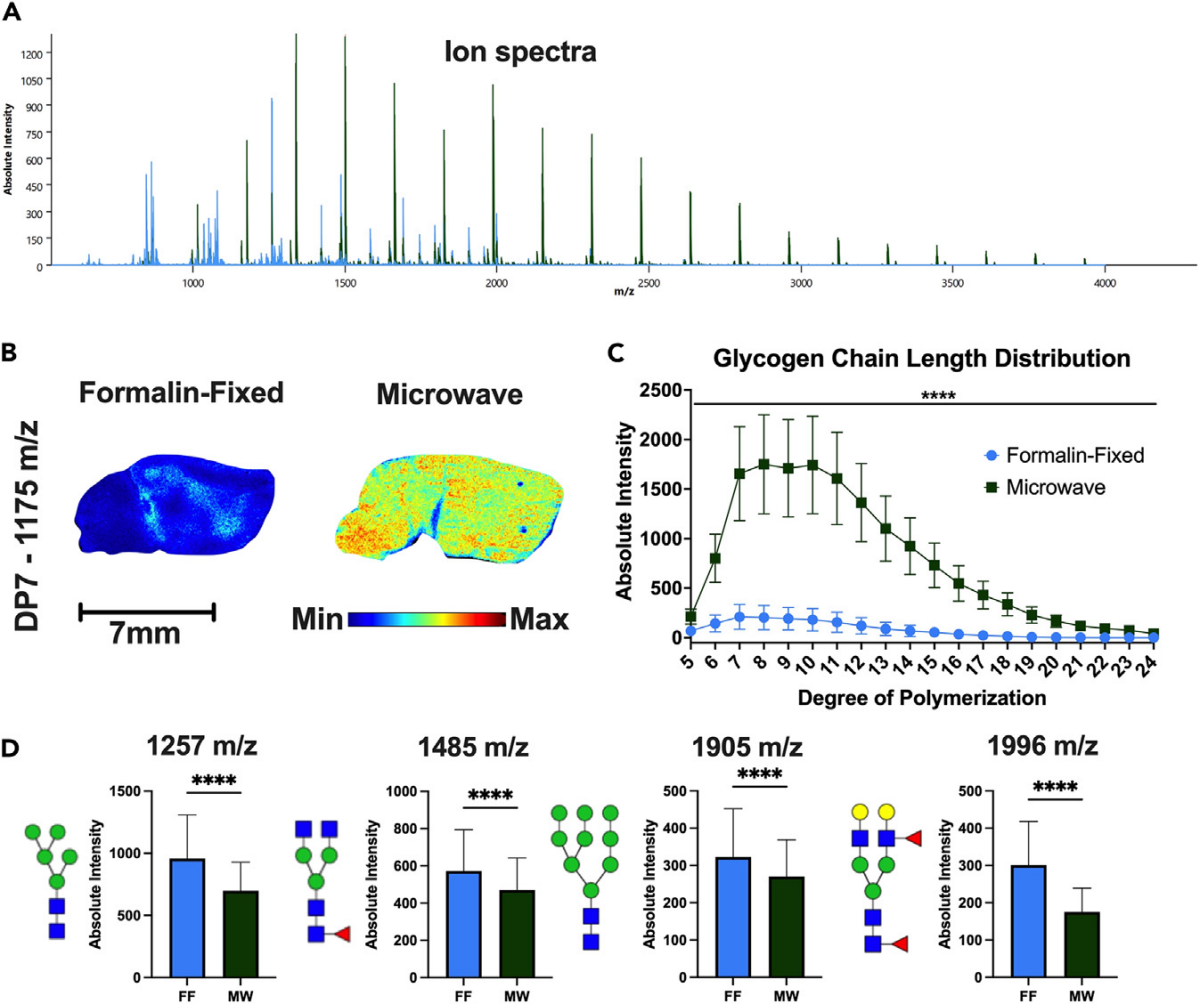
Glycogen and N-linked glycan levels from microwave- and formalin-fixed brain tissue quantified by MALDI-MSI (A) Representative ion mass spectra from MALDI-MSI. (B) Representative images from MALDI-MSI for localization of chain length 7, i.e., degree of polymerization. Size and color scales displayed below images. (C) Glycogen chain length distribution from the frontal cortex of microwave- and formalin-fixed tissue. (D) Absolute intensity of selected N-linked glycans in the frontal cortex for microwave- and formalin-fixed tissue. Values are presented as mean +/− standard deviation (*n* = 3,443 pixels for formalin-fixed brain, *n* = 2,868 pixels for microwave-fixed brain). All pixels are from one representative biological replicate for each treatment. ∗∗∗∗ = *p* < 0.0001.

**Figure 4:**
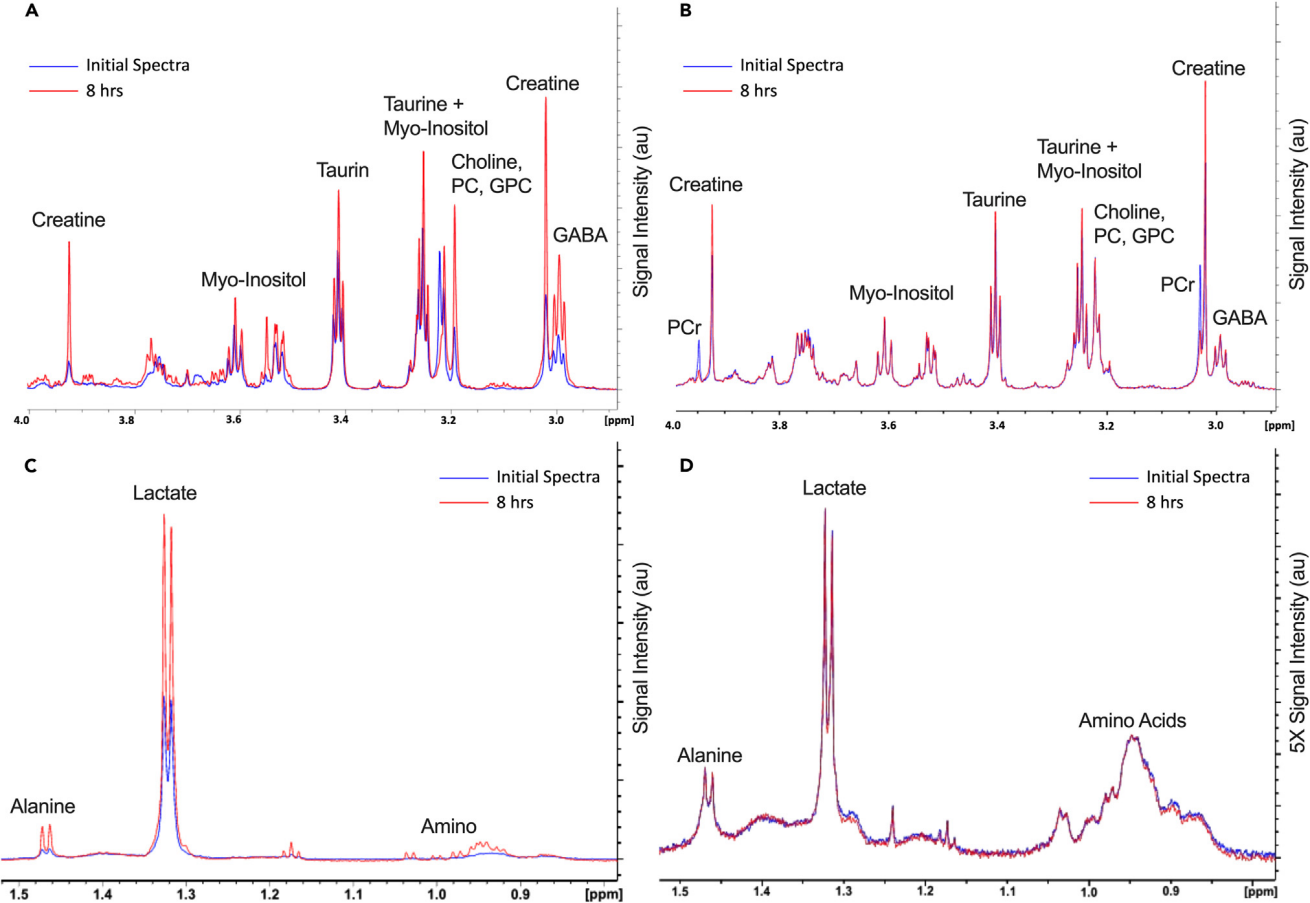
Expected protein degradation and metabolic changes post-euthanasia Tissue was kept at 4°C and acquired using a HRMAS NMR probe at 800 MHz. (A) NMR spectra for fresh frozen tissue after 20 min and 8 h displaying small metabolites (PC = phosphocholine; GPC = glycerophosphocholine). (B) NMR spectra for microwave-fixed tissue after 20 min and 8 h displaying small metabolites (PCr = phosphocreatine). (C) Representative NMR spectra showing lactate and alanine levels for fresh frozen tissue at 20 min and 8 h. (D) Representative NMR spectra showing lactate and alanine levels for microwave-fixed tissue at 20 min and 8 h.

Brain biochemistry is sensitive both to the method of euthanasia and the time needed to fix the tissue. Tissues are subjected to hypoxic conditions after euthanasia that drastically change molecular processes, such as post-translational modification of proteins.^4^ Consequently, hypoxia affects metabolic processes by altering signaling and reducing the oxygen availability for oxidative phosphorylation, ultimately resulting in rapid changes in carbon metabolism.^5^^,^^6^ Unsurprisingly, brain glycogen levels are also sensitive to the euthanasia method and fixation time, rapidly changing during and after common euthanasia methods, e.g., anesthesia and CO_2_ asphyxiation.^7^^,^^8^^,^^9^^,^^10^^,^^11^ In fact, brain glycogen levels are reduced by approximately 50% only 1 min after decapitation.^12^ To test the preservation effectiveness of the microwave, we compared glycogen levels in brain tissue from mice euthanized by cervical dislocation and decapitation followed by freezing versus brain tissue from mice euthanized by high-power microwave. Once cryo-preserved, tissue was fixed in formalin and prepared for glycomic analysis by MALDI-MSI using a well-established protocol (Figure 3).^13^^,^^14^^,^^15^ As predicted, brain glycogen levels were significantly higher in microwave-fixed tissue (Figures 3B and 3C). Using MALDI-MSI, we were also able to determine the effects of the fixation method on N-linked glycan abundance. Surprisingly, we observe a marked decrease in N-linked glycan abundance using the microwave-fixed tissue compared to formalin-fixed tissue (Figure 4D). The change in glycan abundance is an example of a post-translational modification that occurs using standard euthanasia methods, i.e., N-linked glycosylation increases during non-microwave euthanasia.

Moreover, to assess additional changes in brain metabolites post-euthanasia, we utilized HRMAS ^1^H-NMR to quantify metabolic changes over time (Figure 4). Spectra from brain tissue from mice sacrificed via cervical dislocation or high-power, brain-focused microwave were acquired using a T_2_ filtered PROJECT sequence to minimize macromolecular background. Small metabolites were more stable after microwave fixation (Figure 4). In fresh frozen tissue, choline levels increased while phosphocholine and glycerophosphocholine decreased while the same metabolites remained unchanged in the microwave-fixed tissue (Figures 4A and 4B). Surprisingly, we are able to detect phosphocreatine in microwave-fixed tissue, which cannot be detected in tissue from fresh frozen tissue (Figures 4A and 4B). Lactate and alanine levels were significantly elevated in tissue collected via cervical dislocation compared to the microwave-fixed tissue (Figures 4C and 4D). Moreover, the signal was stable for microwave-fixed tissue while tissue progressively degraded in the fresh frozen tissue (Figures 4C and 4D).

## Limitations

Microwave fixation utilizes heat to inactivate proteins and prevent metabolic changes due to enzymatic reactions. However, certain metabolites may be chemically modified or degraded as a consequence of the high temperature, such as the degradation of adenosine triphosphate to adenosine monophosphate and the conversion of inosine to hypoxanthine.^16^ Although large-scale heat-induced chemical modifications are unlikely to occur due to the short exposure, researchers studying sensitive metabolites should take this into consideration if choosing to use microwave fixation.

The described protocol has been tested and confirmed in animals that have not undergone a craniotomy or other major CNS-centric procedures. It is likely that a craniotomy or other major CNS-related surgery would compromise the structural integrity of the tissue and thus result in overheating or underheating the tissue.

The microwave beam is fine-tuned to fix the brain and subsequently analyze brain metabolism. While fixing the heart using this system has been demonstrated,^17^ a recent study using high-powered, brain-focused microwave euthanasia identified only minor metabolic differences in the liver and skeletal muscle of microwave euthanized animals versus cervical dislocation and decapitation.^1^ Therefore, fixation of peripheral tissues will require optimization.

Brain metabolism is sensitive to pre-euthanasia stimuli, including handling and stress.^6^^,^^18^^,^^19^ Changes in brain metabolism due to different forms of stress may need to be accounted for.

## Troubleshooting

### Problem 1

In the “dissection” section, the tissue is under or over-fixed.

### Potential solution

The weight, genotype, and/or lipid and water content of the animal will greatly influence the fixation quality of the brain tissue. Thus, it is necessary to perform optimization experiments for each genotype and body weight prior to initiating the study.

### Problem 2

In the “place the animal into the high-power, brain-focused microwave” section, rapid vaporization of urine in the bladder causes bladder perforation and significant internal damage.

### Potential solution

Ensure mouse urinates prior to microwave fixation.

### Problem 3

In the “dissection” section, structural integrity of the brain is not maintained, even after optimization.

### Potential solution

Some mouse models may be incompatible with microwave fixation. For example, models that require surgical procedures that impair the integrity of the skull, e.g., controlled cortical impact, may result in damage to the structure of the brain by vapor expulsion.

### Problem 4

In the “placing the animal into the holder” section, animals are difficult to place in the animal holder.

### Potential solution

Scruffing the animals and placing the head in the animal holder generally helps prevent escape. The plunger can then be used to very gently push the animal into the holder. Do not use excessive force and be sure the tail is placed correctly through the plunger to ensure the animal experiences no pain.

### Problem 5

In the “place the animal into the high-power, brain-focused microwave” section, the animal survived the microwave irradiation.

### Potential solution

Although unlikely, if the animal were to survive the procedure, it would be unconscious and must be sacrificed by a secondary euthanasia method (e.g., cervical dislocation and decapitation).

## Resource availability

### Lead contact

Further information and requests for resources and reagents should be directed to and will be fulfilled by the lead contact, Matthew S. Gentry (matthew.gentry@ufl.edu).

### Technical contact

Technical questions on executing this protocol should be directed to and will be answered by the technical contact, Alex Cantrell (alexrcantrell@gmail.com).

### Materials availability

This study did not generate new unique reagents.

### Data and code availability

No new code was generated for these analyses. Primary data are available upon request.

## Acknowledgments

This study was supported by the 10.13039/100000002National Institute of Health (NIH) grants R01AG066653, R01CA266004, R01AG078702, RM1NS133593, and R01CA288696 to R.C.S. and R35NS116824 and R33NS111081 to M.S.G.; NIH grants R01DK122811 and R01NS092552 to B.N.S.; and NIH NS102196 and 10.13039/100012645Kentucky Spinal Cord and Head Injury Research Trust 22-1A to D.A.A. L.E.A.Y. was supported by NIH/NCI F99CA264165. This research was also supported by funding from the 10.13039/100015087University of Kentucky Markey Cancer Center and the NIH-funded Biospecimen Procurement and Translational Pathology Shared Resource Facility, as well as the Cancer Research Informatics Shared Resource Facility, of the 10.13039/100015087University of Kentucky Markey Cancer Center (P30CA177558). A portion of this work was performed in the McKnight Brain Institute at the National High Magnetic Field Laboratory’s Advanced Magnetic Resonance Imaging and Spectroscopy (AMRIS) Facility, which is supported by 10.13039/100000001National Science Foundation Cooperative Agreement DMR-2128556 and the State of Florida.

## Author contributions

R.C.S., M.S.G., J.R.L., C.W.V.K., D.A.A., and B.N.S. conceptualized the study and designed the experimental workflow. T.M. and R.A.R. prepared samples for and operated the MALDI-MSI. J.C., A.A.B., and J.R.L. performed ^1^H-NMR and analyzed data. J.A.J. and L.E.A.Y. optimized the protocol and performed experiments. A.R.C., T.R.H., J.A.J., and L.E.A.Y. generated figures. A.R.C., T.R.H., J.A.J., M.S.G., and R.C.S. wrote the manuscript. All authors read and approved the manuscript.

## Declaration of interests

R.C.S. is a member of the Medical Advisory Board for Little Warrior Foundation. M.S.G. has research support and research compounds from Maze Therapeutics, Valerion Therapeutics, and Ionis Pharmaceuticals. M.S.G. also received consultancy fees from Maze Therapeutics, PTC Therapeutics, and the Glut1-Deficiency Syndrome Foundation.
